# A complex network of additive and epistatic quantitative trait loci underlies natural variation of *Arabidopsis thaliana* quantitative disease resistance to *Ralstonia solanacearum* under heat stress

**DOI:** 10.1111/mpp.12964

**Published:** 2020-09-11

**Authors:** Nathalie Aoun, Henri Desaint, Léa Boyrie, Maxime Bonhomme, Laurent Deslandes, Richard Berthomé, Fabrice Roux

**Affiliations:** ^1^ LIPM Université de Toulouse INRAE CNRS Castanet‐Tolosan France; ^2^ SYNGENTA seeds Sarrians France; ^3^ LRSV Université de Toulouse CNRS Université Paul Sabatier Castanet‐Tolosan France

**Keywords:** epistasis, GWA mapping, heat stress, local score, natural accessions, *Ralstonia solanacearum*, SOLO DANCER

## Abstract

Plant immunity is often negatively impacted by heat stress. However, the underlying molecular mechanisms remain poorly characterized. Based on a genome‐wide association mapping approach, this study aims to identify in *Arabidopsis thaliana* the genetic bases of robust resistance mechanisms to the devastating pathogen *Ralstonia solanacearum* under heat stress. A local mapping population was phenotyped against the *R. solanacearum* GMI1000 strain at 27 and 30 °C. To obtain a precise description of the genetic architecture underlying natural variation of quantitative disease resistance (QDR), we applied a genome‐wide local score analysis. Alongside an extensive genetic variation found in this local population at both temperatures, we observed a playful dynamics of quantitative trait loci along the infection stages. In addition, a complex genetic network of interacting loci could be detected at 30 °C. As a first step to investigate the underlying molecular mechanisms, the atypical meiotic cyclin *SOLO DANCERS* gene was validated by a reverse genetic approach as involved in QDR to *R. solanacearum* at 30 °C. In the context of climate change, the complex genetic architecture underlying QDR under heat stress in a local mapping population revealed candidate genes with diverse molecular functions.

## Graphical abstract

A genome‐wide association study of the natural variation of *Arabidopsis thaliana* response to *Ralstonia solanacearum* under heat stress revealed resilient quantitative disease resistance underlain by a complex genetic network of additive and epistatic quantitative trait loci.

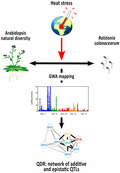

## 1 INTRODUCTION

Climate scenarios predict that extreme climate events will become more frequent by the end of the century (IPCC, 2018), alongside an expected increase in global surface temperature from 1.5 to 4.8°C (IPCC, 2018). In such a context of climate warming, global food security is at risk, with crop yields threatened by both the direct effect of increased temperature on plant development (Hatfield *et al*., 2011; Saidi *et al*., 2011; Bita and Geratz, 2013; Gray and Brady, 2016) and the indirect effect of increased temperature on the emergence of new pathogens and the number and severity of epidemics (Garett *et al*., 2006; Evans *et al*., 2008; Bebber *et al*., 2013; Bita and Gerats, 2013; Suzuki *et al*., 2014). Unravelling the genetic and molecular mechanisms allowing plants to face pathogen attacks under elevated temperature therefore represents a promising strategy for sustainable disease resistance.

Being sessile, plants have developed a wide range of immune responses to face simultaneous and/or sequential stresses caused by various bioaggressors (Roux and Bergelson, 2016). Plant immunity relies on a surveillance system involving plasma membrane‐anchored pattern recognition receptors (PRRs) that perceive microbial elicitors, called pathogen‐ or microbe‐associated molecular patterns (PAMPs or MAMPs). PRR‐triggered immunity (PTI) is efficient against a broad spectrum of pathogens (Cook *et al*., 2015). Adapted pathogens such as phytopathogenic bacteria trigger susceptibility thanks to secreted virulence factors called effectors that can inhibit PTI and promote pathogen invasion (effector‐triggered susceptibility, ETS). The specific recognition of pathogen effectors by plant intracellular nod‐like receptors (NLRs) triggers a more robust immune response called effector‐triggered immunity (ETI), often associated with a cell death response or hypersensitive response (HR) that restricts pathogen invasion to the infection site. In general, ETI is specific to a single pathogenic species, and even to a single pathogenic strain. This specificity causes a strong selective pressure on virulent strains to bypass ETI, making in most cases this form of immunity not durable in crop field conditions (Roux *et al*., 2014). Another form of resistance represented by a reduction rather than an absence of disease refers to quantitative disease resistance (QDR) (St Clair, 2010; Mundt, 2014; Roux *et al*., 2014; French *et al*., 2016). QDR is generally polygenic, durable and broad spectrum (Young, 1996; Poland *et al*., 2009). Unlike PTI and ETI, molecular mechanisms underlying QDR remain largely unknown (Roux *et al*., 2014). Noteworthy is the alteration of all these major forms of immunity by heat stress. Numerous studies involving various pathosystems reported inhibition of ETI responses by a temperature increase (3–7°C) (de Jong *et al*., 2002; Xiao *et al*., 2003; Yang and Hua, 2004; Wang *et al*., 2009; Cheng *et al*., 2013; Menna *et al*., 2015; Aoun *et al*., 2017; Venkatesh and Kang, 2019).

Bacterial wilt, caused by the gram‐negative bacterium *Ralstonia solanacearum,* is one of the most devastating bacterial diseases in the world. Indeed, this soilborne pathogen affects more than 200 species, including members of Solanaceae and Brassicaceae, and is responsible for dramatic yield losses not only in tropical and subtropical areas, but also in warm temperate regions (Elphinstone, 2005). In the model plant *Arabidopsis thaliana*, a broad‐spectrum resistance response to *R. solanacearum* is conferred by the *RPS4*/*RRS1* *‐R* locus that encodes a pair of NLR receptors cooperating molecularly to form homodimers (Deslandes *et al*., 2002; Birker *et al*., 2009; Narusaka *et al*., 2009; Williams *et al*., 2014). In addition, the leucine‐rich repeat receptor‐like kinase ERECTA was identified as underlying one of the three quantitative trait loci (QTLs) detected against the *R. solanacearum* strain 14.25 (Godiard *et al*., 2003).

The genetic architecture and the molecular mechanisms of plant responses to *R. solanacearum* in changing abiotic environments, and more particularly under elevated temperature conditions, remain elusive. Recently, a genome‐wide association study (GWAS) performed in *A. thaliana* and aimed at exploring the genetic bases associated with the natural variation of plant response to strain GMI1000 at 30°C led to the identification of the Strictosidine Synthase‐Like protein 4 (*SSL4*) gene, although the underlying molecular mechanisms are still unknown (Aoun *et al*., 2017). This study was based on 176 accessions of *A. thaliana* from a worldwide collection. While being informative, a limitation of this mapping population‐based approach resides in an increased effect of the demographic history on genotype–phenotype association at large geographical scales. Statistical methods controlling for confounding by population structure can reduce the rate of false‐positive associations, but to the detriment of a loss of detection power (i.e., markers linked to causative genes that are lost after correcting for population structure; Bergelson and Roux, 2010, Brachi *et al*., 2010). In addition, because different QTLs and/or different alleles at the same QTL can be responsible for the same phenotypic values, the power of GWAS can be strongly reduced by the effects of genetic and allelic heterogeneity due to the increased probability of the presence of rare alleles at large geographical scales (Bergelson and Roux, 2010). To limit these drawbacks, GWA mapping can be combined with traditional linkage mapping (based on the use of experimental populations such as recombinant inbred lines, RILs), which is prone to identifying rare alleles and not subjected to the effect of population structure (Bergelson and Roux, 2010). Combining GWA mapping and traditional linkage mapping has been demonstrated to reduce the rates of false positives and negatives when applied to flowering time data in *A. thaliana* (Brachi *et al*., 2010), but remains time‐consuming due to the need to phenotype thousands of experimental lines. To limit the drawbacks of GWA mapping performed at a worldwide scale, an alternative approach is to work at a small geographical scale (Bergelson and Roux, 2010). As reported in a GWAS performed on flowering in *A. thaliana* from a worldwide to a local scale (by using two highly polymorphic French mapping populations), a great reduction of confounding by population structure was observed at the smaller geographical scales (Brachi *et al*., 2013). In addition, the genetic architecture was highly specific on the considered geographical scale (Brachi *et al*., 2013).

In the present study, we therefore investigated the genetic bases of QDR to *R. solanacearum* under elevated temperature by performing a GWAS at a small geographical scale using the TOU‐A local mapping population. This local mapping population offers several advantages, including (a) the detection of more than 1.9 million single nucleotide polymorphims (SNPs), only 5.6 times less than observed in a panel of 1,135 accessions collected at the worldwide scale (Frachon *et al*., 2017); (b) an extensive genetic variation for a large range of phenotypic traits, including QDR to the bacterial vascular pathogen *Xanthomonas campestris* pv. *campestris*; (c) a linkage disequilibrium (LD) decay below 3 kb allowing fine‐mapping of genomic regions associated with phenotypic variation; (d) a strongly reduced confounding effect by population structure; and (e) an adaptation to local warming in fewer than eight generations (Brachi *et al*., 2013; Huard‐Chauveau *et al*., 2013; Baron *et al*., 2015; Debieu *et al*., 2016; Frachon *et al*., 2017).

Interestingly, this work revealed a genetic architecture of natural variation of QDR to *R. solanacearum* that totally differs from the one previously described at the worldwide scale (Aoun *et al*., 2017). In particular, at 30°C, we observed a playful dynamics of 12 QTLs, showing an increase or a decrease in significance, along the disease symptom progression, with most QTLs displaying complex epistatic relationships. Using a reverse genetic approach, we identified SOLO DANCERS (*SDS*) encoding a cyclin‐like protein as the gene underlying one of the two additive QTLs detected at 30 °C.

## 2 RESULTS

### 2.1 Impact of temperature on genetic variation for QDR to *R. solanacearum* among local *A. thaliana* accessions

**FIGURE 1:**
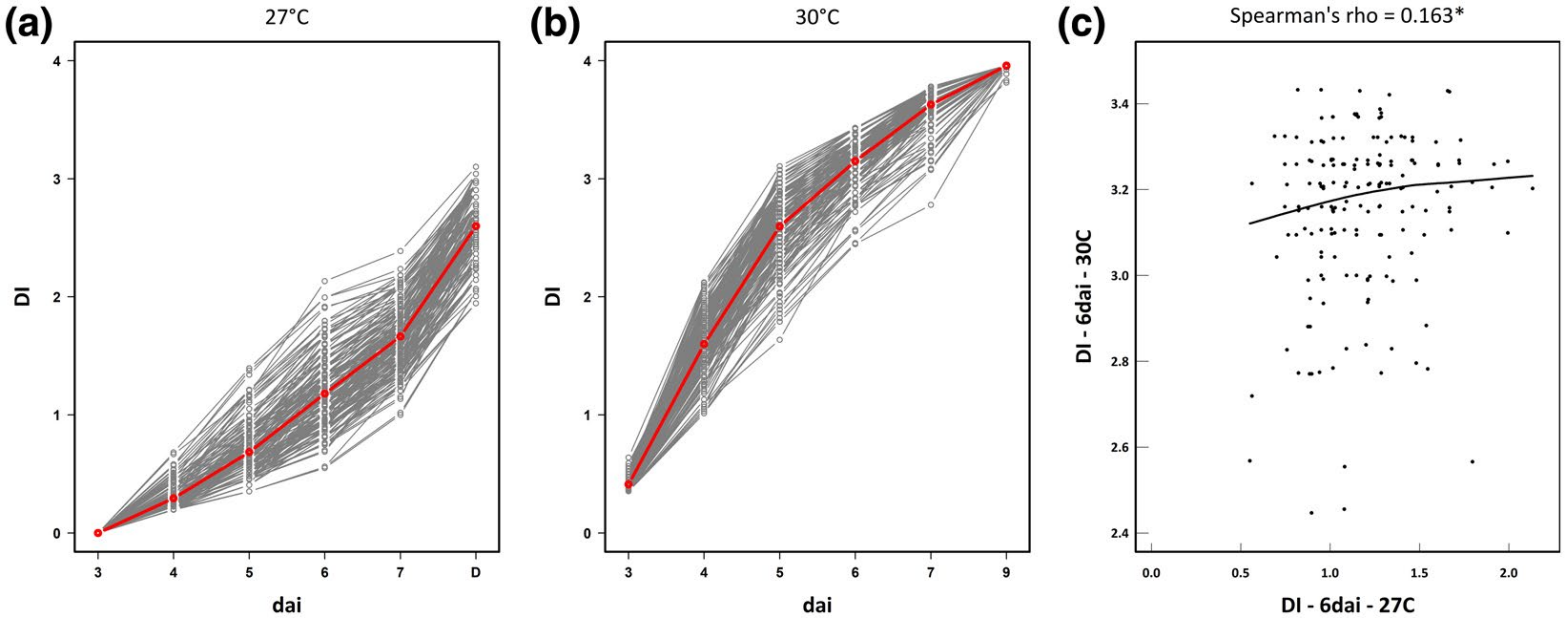
Genetic diversity of plant response to *Ralstonia solanacearum* GMI1000 in the local TOU‐A mapping population. (a) Genetic variation of response dynamics at 27 °C. (b) Genetic variation of response dynamics at 30 °C. The red line represents the mean of disease index over all the accessions in (a) and (b). (c) Relationship between disease index at 6 dai scored at 27 and 30°C. dai, days after inoculation. DI, disease index. The black line represents the locally weighted polynomial regression

In this study, we tested 192 whole‐genome sequenced local accessions of *A. thaliana* in response to the *R. solanacearum* GMI1000 reference strain, under growth chamber conditions. No germination was observed for six accessions that were therefore discarded from the study (Table S1). The remaining 186 local accessions from the TOU‐A population were challenged with GMI1000 at 27 and 30 °C by cutting the roots. The accessions were on average more susceptible at 30 °C than at 27 °C (Figure 1a,b). For each temperature treatment, we observed a large genetic variation at most infection stages, that is, 5, 6, and 7 days after inoculation (dai) at 27 °C and 4, 5, 6, and 7 dai at 30 °C (Table 1 and Figure 1), with broad‐sense heritability estimates ranging from 0.34 to 0.41 at 27°C and from 0.29 to 0.39 at 30 °C (Table 1). Based on genotypic values estimated for the 186 TOU‐A accessions, cross‐temperature genetic correlation was weak, albeit significant (5 dai: Spearman's rho = 0.23, *p* = .003, 6 dai; Spearman's rho = 0.16, *p* = .033, 7 dai; Spearman's rho = 0.20, *p* = .008; Figure 1c), suggesting a flexible genetic architecture of *A. thaliana* response to the GMI1000 strain between 27 °C and 30 °C.

**TABLE 1:** Natural variation among TOU‐A natural accessions for disease index at 27 and 30°C

Temperature (°C)	Model terms	Symptoms 3 dai		Symptoms 4 dai		Symptoms 5 dai		Symptoms 6 dai		Symptoms 7 dai		Symptoms 9 dai	
		*F* or LRT	*p*	*F* or LRT	*p*	*F* or LRT	*p*	*F* or LRT	*p*	*F* or LRT	*p*	*F* or LRT	*p*
27	Block	0.7	0.5262	1.7	0.2049	6.3	0.0036	36.4	0.0002	58.4	0.0002	50.9	0.0002
	*Accession*	0.0	1.0000	2.9	0.1076	9.0	0.0042	14.3	0.0004	7.9	0.0069	3.3	0.0906
	Control Col‐0	ne	ne	54.8	0.0002	105.9	0.0002	122.8	0.0002	110.0	0.0002	87.1	0.0002
	*H* ^2^	0.00^ns^		0.21^ns^		0.35**		0.41***		0.34**		0.37^ns^	
30	Block	3.7	0.0382	20.9	0.0004	18.2	0.0004	9.0	0.0004	5.8	0.0064	4.2	0.0602
	*Accession*	1.4	0.2874	9.5	0.0051	18.4	0.0004	8.7	0.0064	12.4	0.0014	0.2	0.6956
	Control Col‐0	0.1	0.7862	12.2	0.0014	6.8	0.0158	0.4	0.5967	2.4	0.1595	ne	ne
	*H* ^2^	0.13^ns^		0.29**		0.39***		0.3**		0.37**		0.11^ns^	

*F*, *F* value resulting from the test of fixed effect; LRT, LRT value resulting from the likelihood ratio test; *H* ^2^, broad‐sense heritability values; dai, days after inoculation; ne, not estimated due to the absence of variation in disease symptoms among Col‐0 control plants; ns, not significant. Italic terms indicate random effects.

** *p* < .01;

*** *p* < .001.

### 2.2 Playful dynamics of QTLs at 27 and 30 °C

**FIGURE 2:**
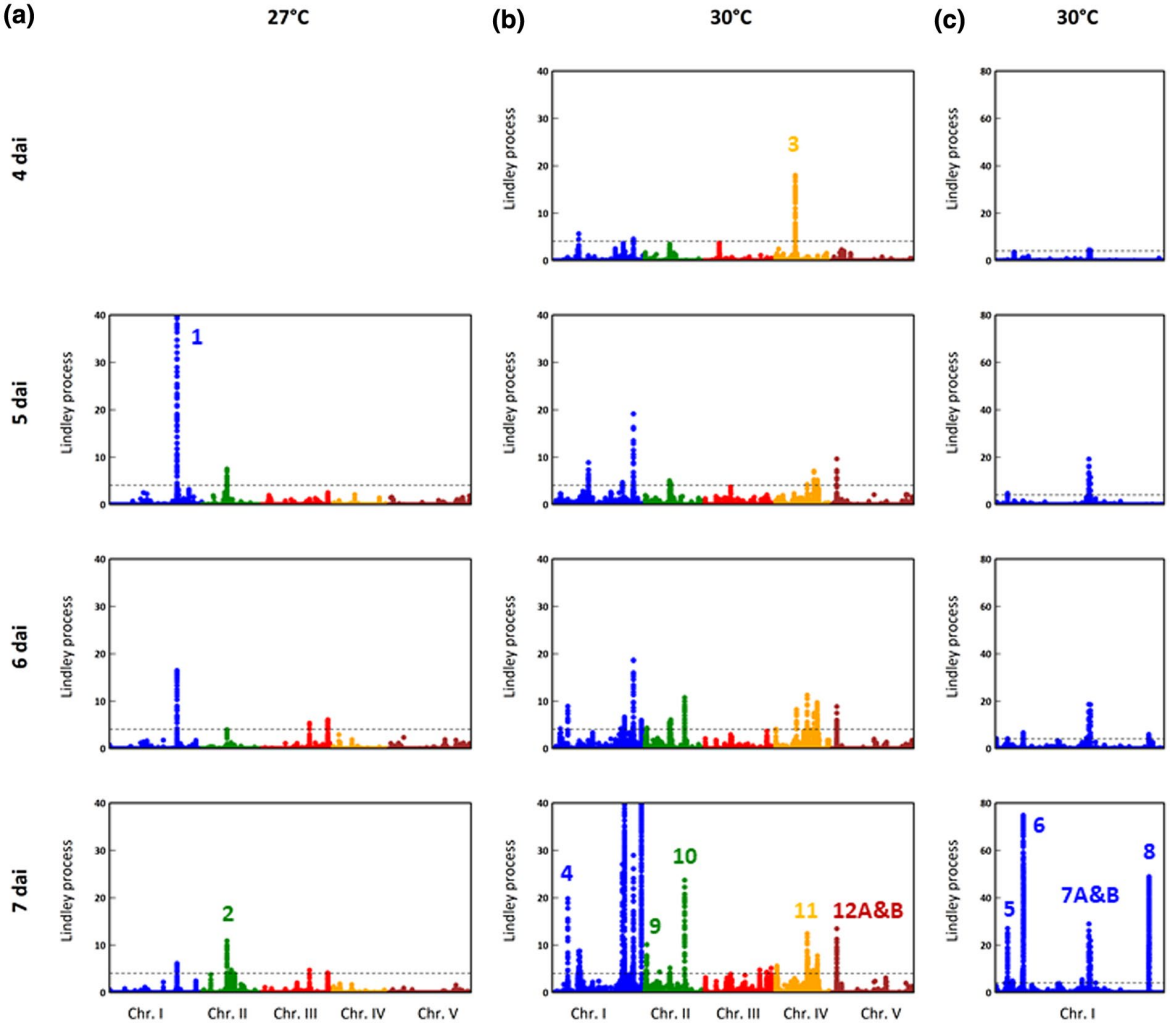
The genetics of quantitative disease resistance to *Ralstonia solanacearum* GMI1000 in the TOU‐A population. (a) Manhattan plot of the Lindley process (local score method with a tuning parameter ξ = 2) at 5, 6, and 7 days after inoculation ( dai) at 27 °C. (b) Manhattan plot of the Lindley process (ξ = 2) at 4, 5, 6, and 7 dai at 30 °C. (c) Zoom spanning a genomic region at the end of chromosome I from 23 Mb to 29.3 Mb containing five quantitative trait loci (QTLs). The dashed line indicates the maximum of the five chromosome‐wide significance thresholds. To better highlight minor QTLs in (a) and (b), Lindley process values on the *y* axis range from 0 to 40. Note that for the main association peak detected on chromosome I at V dai and 27 °C, the highest local score value is 58.6. The number close to association peaks correspond to the 14 QTLs with a Lindley process value above 10. "7A&B" corresponds to two QTLs on chromosome I separated by c.63.5kb. "12A&B" corresponds to two QTLs on chromosome V separated by c.27.1kb

**FIGURE 3:**
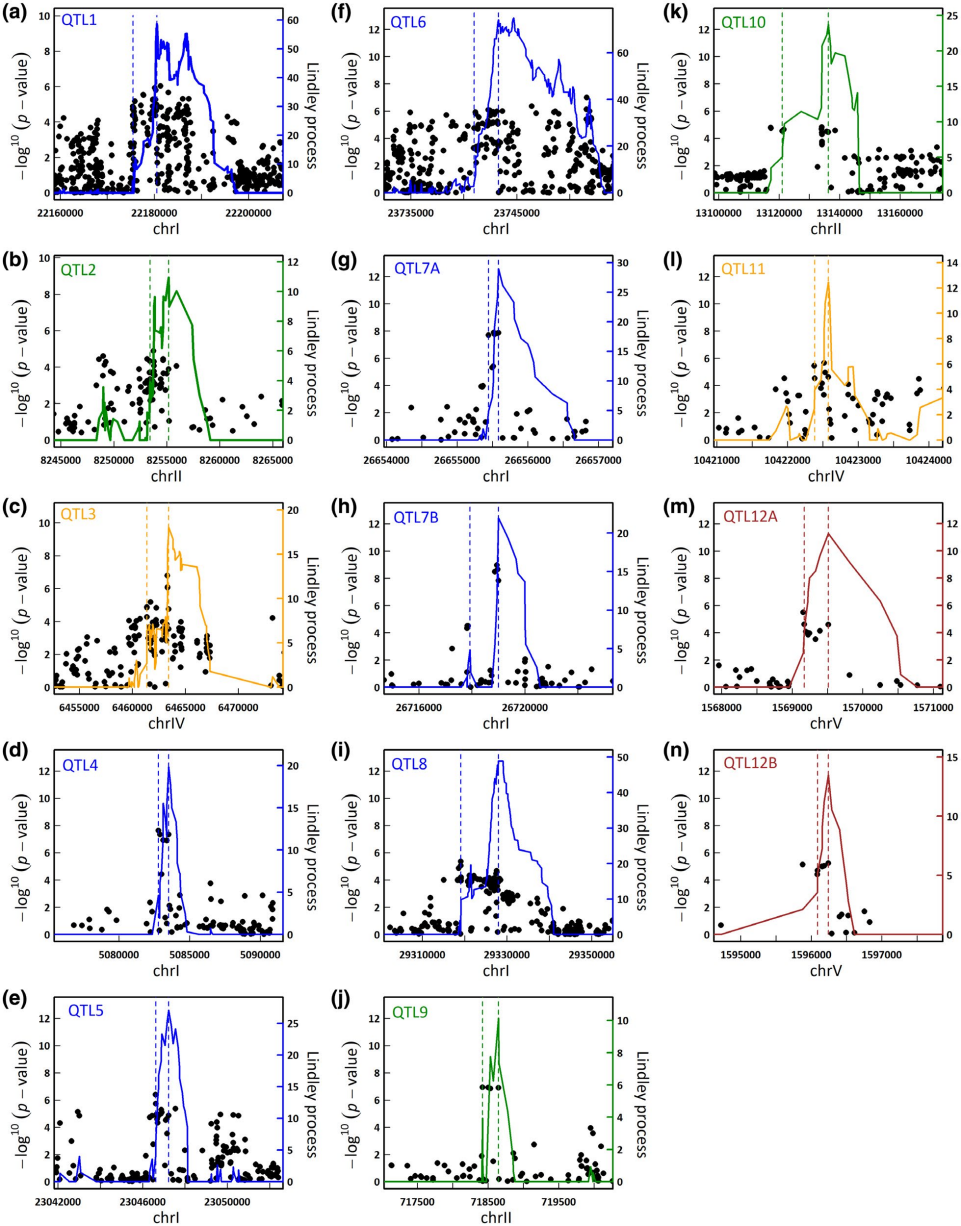
Zoom spanning the 14 quantitative trait loci (QTLs) with a Lindley process value above 10. Each of the 14 QTLs highlighted in Figure 2 are depicted from (a) to (n). The *x* axis corresponds to the physical position of the single nucleotide polymorphisms (SNPs). The dots correspond to the −log_10_ *p* values of the SNPs obtained with the mixed model implemented in the EMMAX software (*y* axis on the left). The solid coloured curve indicates the Lindley process (local score method with ξ = 2) calculated from left to right (*y* axis on the right). The two coloured dashed vertical lines indicate the QTL intervals detected, without taking into account the right part of the curve (Fariello *et al*., 2017; Bonhomme *et al*., 2019)

To increase the probability to discover QTLs with additive effects conferring QDR to *R. solanacearum* along the infection stages, we combined a genome‐wide association mapping approach with a local score analysis (with tuning parameter ξ = 2) (Bonhomme *et al*., 2019). We detected over the infection stages 215 and 738 significant unique SNPs (i.e., top SNPs) at 27 and 30 °C, respectively (Figure 2). In agreement with weak cross‐temperature genetic correlation, no single top SNP was common to both temperatures, indicating a contrasted genetic architecture for natural variation of response to *R. solanacearum* GMI1000 between 27 and 30 °C. Next, we focused on the 14 most highly significant additive QTLs (i.e., top QTLs with a Lindley process >10, Figures 2 and 3). Two top QTLs were detected at 27 °C while the remaining top QTLs were detected at 30 °C (Figure 2). Interestingly, all these top QTLs displayed playful dynamics, with two QTLs (i.e., QTL1 at 27 °C and QTL3 at 30 °C) and 12 QTLs showing a decrease and increase in significance with advanced infection stages, respectively (Figure 2).

**FIGURE 4:**
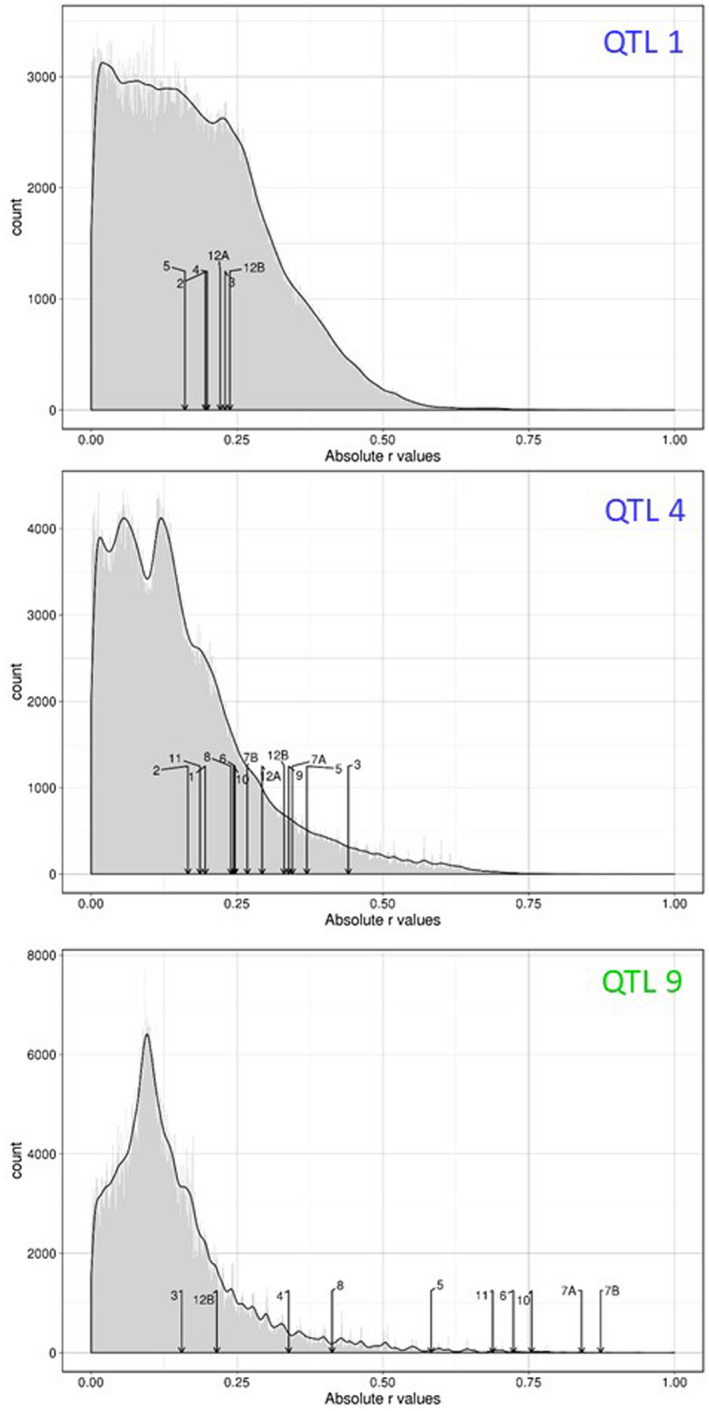
Detection of inter‐QTLs epistasis for QTL1 (a), QTL4 (b), and QTL9 (c). For each quantitative trait locus (QTL), a genome‐wide distribution (grey area) was established by calculating linkage disequilibrium (LD) values between the bait top single nucleotide polymorphism (SNP) and all the other SNPs across the genome (with the exception of the SNPs located in a 100 kb window surrounding the bait top SNPs). Only SNPs with a minor allele relative frequency (MARF) > 0.07 were considered. In addition, LD values (above 0.1) between the bait top SNP for the corresponding QTL and the bait top SNPs from the other QTLs are represented by arrows. The *x* axis corresponds to the LD estimates expressed as the absolute value of the *r* correlation coefficient. The black line corresponds to the density curve

**FIGURE 5:**
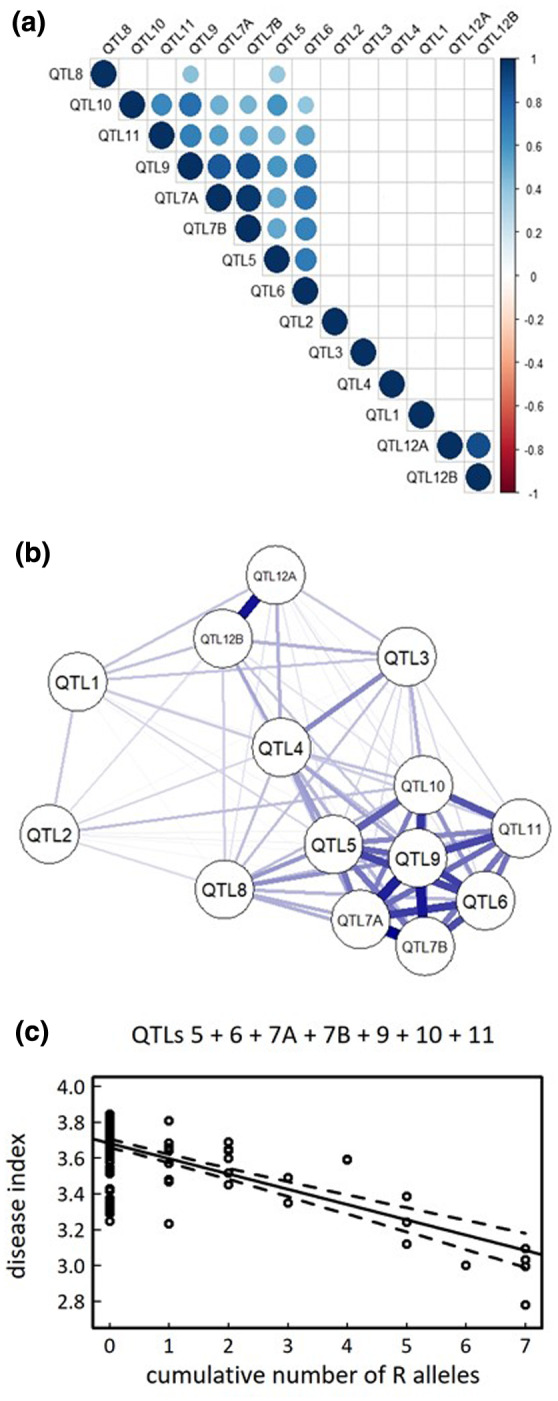
Linkage disequilibrium (LD) patterns among the 14 quantitative trait loci (QTLs) with a Lindley process above 10. (a) Graphical display of the LD matrix. (b) Weighted network visualization of the LD matrix (*qgraph* library implemented in the *R* environment). (c) Relationship between disease index and the cumulative number of resistant R alleles at the seven QTLs of the first group of epistatic QTLs. Dots correspond to the kinship‐adjusted genotypic values of the TOU‐A accessions. A total amount of 47.1% of disease index variation was explained by the cumulative number of resistance alleles at the seven QTLs

Based on LD calculation among the 14 top QTLs, both QTLs detected at 27 °C only present additive (i.e., independent) effects (Figures 4, S1, and S2). By contrast, at 30 °C, nine out of the 12 top QTLs also displayed epistatic interactions (Figures 4 and S2) with the identification of two groups of epistatic QTLs (Figure 5). The first one regroups seven QTLs (QTL5 + QTL6 + QTL7A + QTL7B + QTL 9 + QTL10 + QTL11) with highly significant pairwise LD values (*p < *.001); in particular at the interchromosomal level (Figures 4, 5, and S2). Based on the representative SNPs of the seven QTLs, 47.1% of disease index variation was explained by the cumulative number of resistance alleles at these QTLs (Figure 5c). It should be noted that c.80% of the accessions have a susceptible allele at each of the seven QTLs (Figure 5c), precluding testing with sufficient power any pairwise interactions among these QTLs. The second group contains QTL12A + QTL12B (Figure 5), with a clear disequilibrium in the number of accessions among the four expected haplotypes (SS = 88.9%, RR = 9%, RS = 0.7%, SR = 1.4%; Figure S1). QTL8 showed weak epistatic relationships with QTL5 and QTL9 (Figure 5a), whereas no significant epistatic relationship was detected for the two remaining QTLs detected at 30 °C (i.e., QTL3 and QTL4) (Figures 5, S1, and S2).

### 2.3 Molecular functions of the candidate gene products underlying the QTLs identified

In agreement with the LD decay below 3 kb observed in the TOU‐A population, the average size of the 14 QTLs was 4,589 bp (min = 141 bp, max = 26.07 kb, Figure 3), thereby limiting the number of candidate genes underlying each QTL. The AGI locus code and the corresponding predicted molecular function(s) of the candidate genes are indicated in Table 2. At 27°C, the top SNP of QTL1 (SNP‐1‐22180112) is located in the coding region of TREHALOSE PHOSPHATE SYNTHASE 10 (*At1g60140*) (Figure 3a). QTL2 covers a short region of c.1.77 kb, with the top SNP SNP‐2‐8253743 located in a gene (*At2g19050*) belonging to a GDSL‐like Lipase/Acylhydrolase protein superfamily (Figure 3b).

**TABLE 2:** List of candidate genes underlying the 14 quantitative trait loci (QTLs) with a Lindley process value above 10 at 27 and 30°C

Temperature (°C)	dai	QTL id	Chromosome	Bait SNP	*p* value	Gene	Gene description
27	5	QTL1	I	22 180 112	1.20 × 10^−6^	***At1g60140***	**TREHALOSE PHOSPHATE SYNTHASE 10**
	7	QTL2	II	8 253 743	1.27 × 10^−5^	***At2g19050***	**GDSL‐like lipase/acylhydrolase superfamily protein**
30	4	QTL3	IV	6 463 310	1.63 × 10^−7^	*At4g10440*	S‐adenosyl‐L‐methionine‐dependent methyltransferases superfamily protein
						***At4g10450***	**Ribosomal protein L6 family**
	7	QTL4	I	5 082 790	2.30 × 10^−8^	***At1g14750***	**SOLO DANCERS**
	7	QTL5	I	23 021 261	2.28 × 10^−8^	*At1g62305*	Core‐2/I‐branching β‐1,6‐*N*‐acetylglucosaminyltransferase family protein
						*At1g62330*	*O*‐fucosyltransferase family protein
	7	QTL6	I	23 742 319	8.08 × 10^−7^	***At1g63980***	**D111/G‐patch domain‐containing protein**
	7	QTL7A	I	26 655 520	1.31 × 10^−7^	***At1g70700***	**JASMONATE‐ZIM‐DOMAIN PROTEIN 9**
	7	QTL7B	I	26 718 947	1.07 × 10^−9^	***At1g70860***	**Polyketide cyclase/dehydrase and lipid transport superfamily protein**
	7	QTL8	I	29 319 094	4.28 × 10^−6^	***At1g77990***	**SULPHATE TRANSPORTER 2**
	7	QTL9	II	718 424	1.15 × 10^−7^	***At2g02620***	**Cysteine/histidine‐rich C1 domain family protein**
	7	QTL10	II	13 134 129	1.51 × 10^−5^	*At2g30800*	HELICASE IN VASCULAR TISSUE AND TAPETUM
						*At2g30810*	Gibberellin‐regulated family protein
						*At2g30820*	Aspartyl/glutamyl‐tRNA(Asn/Gln) amidotransferase subunit
						***At2g30830***	**2‐Oxoglutarate (2OG) and Fe(II)‐dependent oxygenase superfamily protein**
						*At2g30840*	2‐Oxoglutarate (2OG) and Fe(II)‐dependent oxygenase superfamily protein
	7	QTL11	IV	10 422 518	2.21 × 10^−6^	***At4g19030***	**NOD26‐like intrinsic protein 1**
	7	QTL12A	V	1 569 170	2.41 × 10^−5^	***At5g05290***	**EXPANSIN A2**
	7	QTL12B	V	1 596 241	5.93 × 10^−6^	***At5g05390***	**LACCASE 12**

For each QTL, the candidate genes corresponding to the top single nucleotide polymorphism (SNP) and the flanking gene are in bold and normal text, respectively. dai, days after inoculation.

At 30 °C, the candidate genes underlying the 12 top QTLs encode for various molecular functions. Functional classification was performed with the Classification Superviewer Tool on the University of Toronto website (http://bar.utoronto.ca/ntools/cgi‐bin/ntools_classification_superviewer.cgi) using the MAPMAN classification as source (Provart and Zhu, 2003) and the list of genes to which top SNPs were associated. In particular, two QTLs correspond to genes involved in abiotic stress signalling pathways, that is, QTL3 with the top SNP SNP‐4‐6463310 and QTL7B with the top SNP SNP‐1‐26718947 falling within *At4g10450* and *At1g70860* encoding a ribosomal protein L6 family and a cytokinin responsive lipid transport protein, respectively (Figure 3c,h and Table 2). QTL4 covers a small region of 730 bp, with the top SNP SNP‐1‐5082790 falling within the promoter region of *SOLO DANCERS* (*At1g14750*) that encodes an atypical meiotic cyclin‐like protein (Figure 3d and Table 2). The top SNPs SNP‐1‐29319094 and SNP‐4‐10422518 of QTL8 and QTL11 were located in the genomic region of *At1g77990* (SULPHATE TRANSPORTER 2;2) and *At4g19030* (NOD26‐LIKE INTRINSIC PROTEIN 1,1), respectively, encoding a sulphate and an aquaporin transporter, respectively (Figure 3i,l and Table 2). QTL12A and QTL12B with the top SNP SNP‐5‐1569170 and SNP‐5‐1596241 are located within genes encoding EXPANSIN A2 (*At5g05290*) involved in cell wall modification and a lignin peroxidase (*At5g05390*) involved in vascular development, respectively (Figure 3m,n and Table 2). However, among the different biological pathways represented by the 18 candidate genes identified, only the hormonal metabolism was significantly overrepresented (*p* < .01). For instance, the top SNPs of QTL7A (SNP‐1‐26655520) and QTL10 (SNP‐2‐13134129) are located in genes *At1g70700* (*JAZ9*) and *At2g30830* (or alongside *At2g30810*), which are involved in jasmonate (JA), ethylene (ET), and gibberellin (GA) hormonal metabolisms, respectively (Figure 3g,h,k and Table 2).

### 2.4 SOLO DANCERS is the gene underlying QTL4 involved in QDR to *R. solanacearum* GMI1000 at 30 °C

**FIGURE 6:**
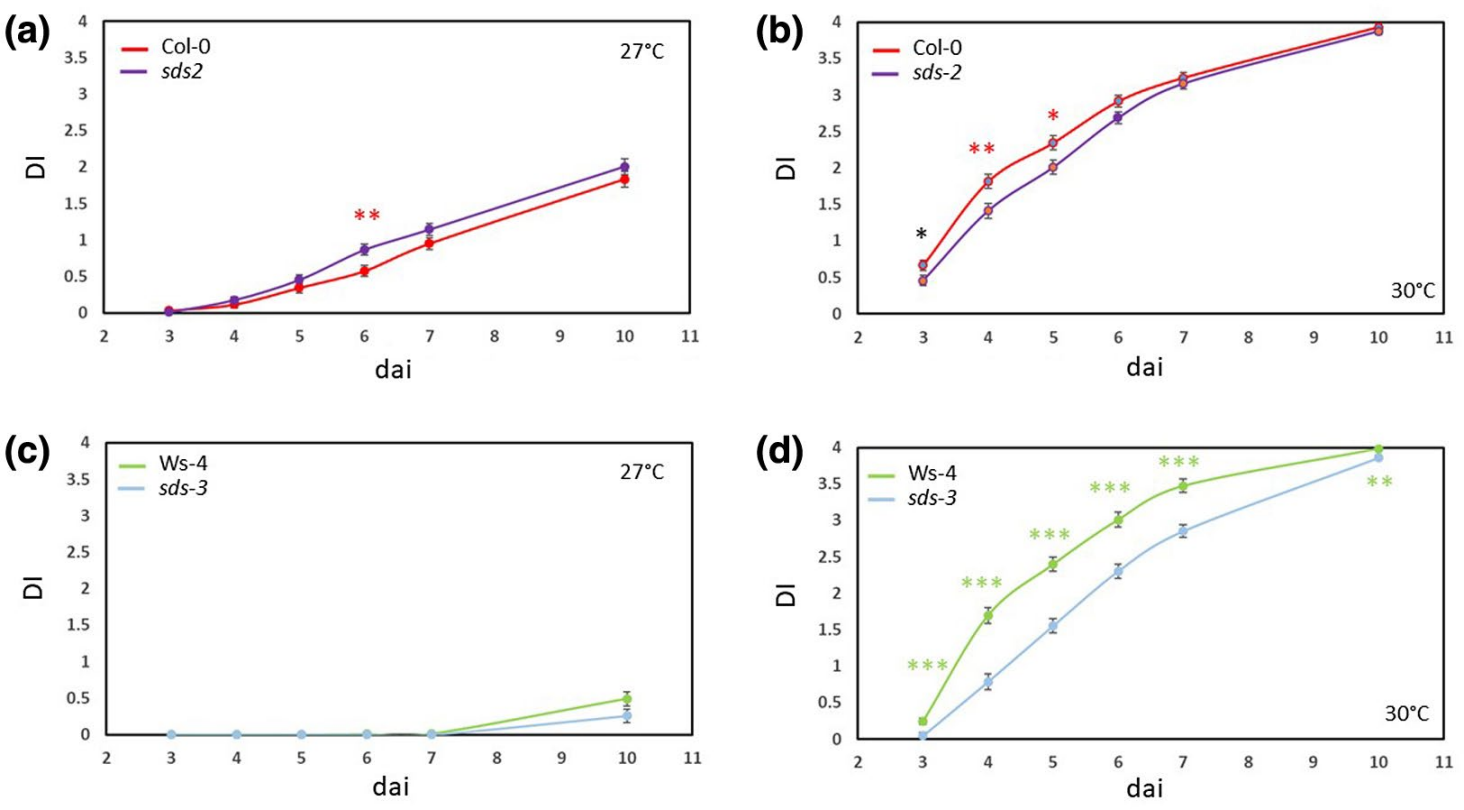
Effects of knockdown of *SDS* expression on the dynamics of disease symptoms after inoculation with the *Ralstonia solanacearum* GMI1000 strain in two genetic backgrounds at 27 and 30 °C. Dynamics of disease symptoms in Col‐0 and *sds‐2* mutant at 27 °C (a) and 30 °C (b). Dynamics of disease symptoms in Ws‐4 and *sds‐3* mutant at 27 °C (c) and 30 °C (d). Least‐square means ± *SE* of the LS means from three independent inoculations (*n* = 72 plants per genetic line × temperature combination. Symbols *, **, and *** denote significant difference observed between each wild‐type background and its corresponding mutant at *p* < .05, *p* < .01, and *p* <.001, respectively. Coloured stars indicate significant differences after a false‐discovery rate correction. DI, disease index; dai, days after inoculation

Next we investigated the molecular mechanisms underlying plant response to *R. solanacearum* at 30 °C in the TOU‐A population. For this, we focused on the additive QTL with the highest allelic effect, that is, QTL4 located on the top of chromosome I and that encompasses the SOLO DANCERS locus (*SDS*, *At1g14750*) (Figures 2b and S1). To check whether *SDS* was involved in this QDR, we monitored at 27 and 30 °C the phenotypical response of *sds‐2* and *sds‐3* null mutants (in Col‐0 and Ws‐4 genetic backgrounds, respectively). Col‐0 and Ws‐4 are both susceptible to GMI1000 at 30 °C while Ws‐4, but not Col‐0, is resistant at 27 °C. At 27 °C, the *sds‐2* mutant response was not significantly different from Col‐0 except at 6 dai (Figure 6a and Table S2). At 30 °C, the wilting of *sds‐2* was significantly delayed compared to that of Col‐0 from 3 to 5 dai (Figure 6b and Table S2). As expected, *sds‐3* mutant and Ws‐4 plants remained symptomless at 27 °C, from 3 to 7 dai (Figure 6c and Table S2). By contrast, at 30 °C, wilting of *sds‐3* plants was strongly reduced during all infection stages (Figure 6d and Table S2). Altogether, these data suggest that *SDS* plays a role in wilting disease development on infection with the *R. solanacearum* GMI1000 strain at 30 °C.

## 3 DISCUSSION

### 3.1 Worldwide versus local genetic variation in *A. thaliana* facing *R. solanacearum*

In comparison with a temperature of 27 °C, local *Arabidopsis* accessions exposed at 30 °C were on average more susceptible to the GMI1000 strain as indicated with a faster wilting disease progression than that observed at 27 °C. This observation is in line with the drastic impact of heat stress on *Arabidopsis* response to the GMI1000 strain previously monitored in a worldwide collection of *A. thaliana* (Aoun *et al*., 2017). This is also in accordance with a growing number of studies performed on crops infected by different pathogenic species that have described the drastic impact of heat stress on resistance response (Moury *et al*., 1998; Jablonska *et al*., 2007; Wang *et al*., 2009; Webb *et al*., 2010). The respective impact of heat stress on host and pathogen is still a matter of debate. Alteration of plant immunity under heat stress has been reported in several studies. For instance, a temperature of 28 °C inhibits “spontaneous lesion” phenotypes or autoimmune responses linked to autoactive alleles of genes encoding NLR resistance proteins (Zhu *et al*., 2010; Negeri *et al*., 2013). While heat stress increases *A. thaliana* susceptibility to infection with *Pseudomonas syringae* pv. *tomato* DC3000, it also promotes the plant‐dependent bacterial multiplication (Huot *et al*., 2017). In this study, *R. solanacearum* grows faster at 30 °C than at 28 °Cunder in vitro conditions (Figure S3a) and a significant increase in bacterial multiplication was observed in planta at 30 °C (Figure S3b), suggesting an effect of heat stress on bacterial multiplication and its pathogenicity.

In previous studies, the level of genetic variation for diverse phenotypic traits such as flowering time and QDR to the bacterial pathogen *X. campestris* was similar between the TOU‐A population and a set of worldwide accessions (Brachi *et al*., 2013; Huard‐Chauveau *et al*., 2013; Debieu *et al*., 2016). Here, the level of phenotypic and genetic variation for QDR to *R. solanacearum* in the TOU‐A population was limited compared to that of the worldwide (WW) collection (mean standard deviation of phenotypic values between 5 and 7 dai: WW 27 °C = 1.61, TOU‐A 27 °C = 1.38, WW 30 °C = 1.08, TOU‐A 30 °C = 0.89; mean *H* ^2^ estimates between 5 and 7 dai: WW 27 °C = 0.77, TOU‐A 27 °C = 0.37, WW 30 °C = 0.74, TOU‐A 30 °C = 0.35; Aoun *et al*., 2017). This may be explained by the absence in the TOU‐A population of fully resistant accessions at 27 °C, which is consistent with the absence of any association peak located around the *RPS4/RRS1‐R* locus on chromosome V (Figure 2). By contrast, this locus was detected as the major association peak at 27 °C in a set of worldwide accessions (Aoun *et al*., 2017).

Given that most of the *A. thaliana* natural populations located in France are genetically diverse (Le Corre, 2005; Platt *et al*., 2010; Brachi *et al*., 2013; Frachon *et al*., 2018; Frachon *et al*., 2019), it would be interesting to investigate the level of genetic variation of QDR to *R. solanacearum* GMI1000 (or to other strains) within those populations. This would provide valuable information on the local dynamics of QDR to a bacterial pathogen in *A. thaliana* at the metapopulation level (Ding *et al*., 2007; Vetter *et al*., 2012; Karasov *et al*., 2014; Roux and Bergelson, 2016).

### 3.2 Complex genetic architecture of QDR to *R. solanacearum* at 27 and 30 °C

Combining GWA mapping related mixed models and genome‐wide local score analysis increases the probability of discovering minor QTLs with additive effects (Fariello *et al*., 2017; Bonhomme *et al*., 2019). This is also well exemplified here with a more detailed characterization of the genetic determinants responsible for QDR to *R. solanacearum*. Because no top SNPs were common to both temperature treatments, our data illustrate how only a weak temperature increase of 3 °C can drastically affect the genetic architecture of QDR to *R. solanacearum*. We next evaluated the effect of the geographic scale on the genetic architecture of this QDR by applying a genome‐wide local score approach to the EMMAX results previously obtained on a set of 176 worldwide *A. thaliana* accessions (Aoun *et al*., 2017). No genomic regions containing top SNPs were shared between the local and worldwide scales (Data S1). Interestingly, similar results were obtained in a study investigating the genetic determinants of flowering time scored on both local and worldwide mapping populations of *A. thaliana* in two environmental conditions simulating two seasonal germination cohorts (Brachi *et al*., 2013). The genetic architecture of flowering was highly dependent on both the geographical scale and the considered season (Brachi *et al*., 2013). Together, the data obtained from various phenotypic traits reinforce the need to account for the geographical scale of phenotypic variation when choosing accession panels for GWAS (Bergelson and Roux, 2010). Consequently, this would help to get a better view of the genetic architecture flexibility of phenotypic traits.

Theoretical predictions suggest that phenotypic changes in ontogenetic time (typically time‐to‐event or time‐to‐failure traits such as flowering time or death time) are often driven by the temporal regulation of QTLs (Johannes, 2007). In this study, all the 14 top QTLs control QDR to *R. solanacearum* GMI1000 in a playful manner at both 27 and 30°C, suggesting that disease progression to *R. solanacearum* highly depends on the time specificity of the genetic effects. At 30°C, disease progression also resulted from a complex genetic network of interacting loci. The more complex genetic architecture observed at 30°C suggests that under heat stress, *A. thaliana* responses to *R. solanacearum* involve a specific and complex network of mechanisms associated with the time specificity of the genetic effects. Few studies analysed the specificities of plant responses to individual or combined stresses, and mostly through transcriptome analyses. Interestingly, it turns out that transcriptional responses of plants to combined stresses are unique and cannot be predicted from that of individual stress (Atkinson and Urwin, 2012; Suzuki *et al*., 2014; Pandey *et al*., 2015). In addition, combined stresses induce a major transcriptional reprogramming characterized by the regulation of the expression of a greater number of genes than observed with individual stresses (Rasmussen *et al*., 2013; Suzuki *et al*., 2014). For instance, more *Arabidopsis* genes were differentially expressed when nematode infection was combined with water stress compared to plants only subjected to nematode infection (Atkinson *et al*., 2013). Similar observations were made in other pathosystems, including *A. thaliana* exposed to the simultaneous application of virus and heat or virus and drought (Prasch and Sonnewald, 2013; Pandey *et al*., 2015). This may indicate that the more complex the environment is, the more the plants establish a response with a polygenetic architecture involving different genetic pathways.

Epistatic networks involving long‐distance LD among physically unlinked loci were reported to represent the main fraction of phenotypic variance for herbivore resistance in *A. thaliana* at a worldwide scale (Brachi *et al*., 2015), yeast growth (Forsberg *et al*., 2017), or body weight and abdominal fat content in chicken (Carlborg *et al*., 2006; Li *et al*., 2013). While complex epistatic relationships among QTLs, including higher‐order epistasis, may be therefore more frequent than anticipated (Carlborg and Haley, 2004; Roux *et al*., 2005; Pettersson *et al*., 2011), the functional validation of epistatic QTLs remains challenging but feasible if we consider a multi‐CRISPR‐Cas9 approach to create double, triple, quadruple, etc. mutants. Nonetheless, it would be interesting to determine whether such an epistatic network is restricted to the TOU‐A population by estimating LD between these five QTLs in other local highly polymorphic populations or at a larger geographical scale.

However, we should stress that some limitations in this study preclude a full description and understanding of the epistatic network underlying QDR to *R. solanacearum*. First, in contrast to traditional mapping populations such as F2 populations or RILs, the number of accessions among the haplotypes expected between two (or more) epistatic QTLs was clearly unbalanced in the TOU‐A population. While it may reflect the maintenance of haplotypes with extreme phenotypes (i.e., susceptible versus resistant) by selective processes (Brachi *et al*., 2015), it precludes testing with sufficient power the magnitude and type of epistasis. Secondly, epistatic relationships were only tested on QTLs with additive effects that were first identified by combining a GWA mapping approach with a local score analysis. Although computationally intensive, a complementary step will be to test the significance of all pairwise interactions among the 981,617 SNPs used in this study, which will require controlling the individual and joint effect of population structure on both SNPs tested in interaction (Wang *et al*., 2018).

### 3.3 Various molecular functions are involved in QDR to the GMI1000 strain at 30°C

Consistent with the molecular functions of previously cloned QDR genes (Roux *et al*., 2014), the nature of most candidate genes underlying the 14 major QTLs identified here is quite diverse and they do not correspond to typical resistance genes encoding NLRs. Indeed, unlike a previous GWAS performed on worldwide *A. thaliana* accessions that led to the detection of the *RPS4/RRS1‐R* NLR locus as the main genetic determinant for full resistance to GMI1000 at 27°C (Aoun *et al*., 2017), the two main QTLs identified at 27°C in the local TOU‐A population do not correspond to any NLR genes. For QTL1, the top SNPs fall in TREHALOSE PHOSPHATE SYNTHASE 10 (*At1g60140*), suggesting that the regulation of trehalose‐6‐phosphate synthesis participates in the plant response. This is consistent with previous data showing that the production of this metabolite by the *R. solanacearum* effector RipTPS plays an important role in pathogen virulence (Poueymiro *et al*., 2014). For QTL2, the top SNPs fall in the *At2g19050* gene encoding a GDSL‐like lipase/acylhydrolase superfamily protein. Interestingly, overexpression of *GLIP1* that also belongs to the *Arabidopsis* GDSL LIPASE‐LIKE gene family was shown to confer enhanced resistance to several pathogens, including *Alternaria brassicicola*, *Erwinia carotovora* and *P. syringae*) (Kwon *et al*., 2009). Therefore, these proteins might also play a role in plant immunity against *R. solanacearum*.

The molecular functions of the candidate genes underlying the 12 major QTLs detected at 30 °C are even more diverse. Interestingly, these functions may reflect different plant responses to face virulence strategies developed by the bacteria to colonize plant tissues and promote its multiplication within the xylem vessels. For instance, candidate genes underlying QTL7A and QTL10 are involved in the synthesis or signalling of hormones that may contribute positively to pathogen resistance and in plant response to combined biotic and abiotic stress. In particular, JA is known to interfere with GA signalling through the degradation of transcriptional repressors such as JAZ9 (the candidate gene underlying QTL7A) to balance plant defence response and growth (Yang *et al*., 2012).

From 4 to 7 dai, QTL4 was detected with increasing significance on chromosome I. The corresponding candidate gene, *SDS*, encodes an atypical meiotic cyclin‐like protein related to A‐ and B‐type cyclins, previously described as being required for DNA double‐strand break (DSB) repair (Azumi *et al*., 2002; De Muyt *et al*., 2009). To our knowledge, *SDS* has never been associated with plant disease susceptibility. Interestingly, two allelic null *sds* mutants were found to be more resistant at both 27 and 30 °C to GMI1000, albeit the allelic effect was different between the two genetic backgrounds. The functional validation of *SDS* as a susceptibility gene represents the first demonstration of its involvement in plant defence response to a bacterial pathogen under heat stress. It is noteworthy that (a) SDS acts together with CYCB3;1 in suppressing unscheduled cell wall synthesis (Bulankova *et al*., 2013); and (b) the two candidate genes underlying QTL12A and QTL12B encode, respectively, proteins involved in cell wall and lignin polymerization. Two cyclin‐L type proteins, MOS12 (Modifier of SNC1, 12) and MOS4‐associated complex (Modifier of SNC1, 4), have also been shown to participate in the alternative splicing of *SNC1* and *RPS4* genes, thereby enabling the fine‐tuning of NLR gene expression (Xu *et al*., 2012). As several NLR genes have been described to be alternatively spliced without knowing the regulatory mechanism (Xu *et al*., 2012), it is tempting to hypothesize that SDS would participate in the regulation of NLR functions under combined *R. solanacearum* and elevated temperature conditions through the production of splicing variants. Because the top SNPs are located in the promoter region of *SDS*, the next step to decipher the underlying molecular mechanisms would be to investigate the natural variation of *SDS* expression in the TOU‐A population and its link to the QDR.

## 4 EXPERIMENTAL PROCEDURES

### 4.1 Bacterial strain, plant material, and growth conditions

The wild‐type *R. solanacearum* GMI1000 strain was grown on complete bacto glucose (BG) agar as previously described (Plener *et al*., 2010). GWAS was performed using 192 whole‐genome sequenced natural accessions of the TOU‐A population (France, Burgundy, 46°38ʹ57.302ʺN, 04°07ʹ16.892ʺE; Frachon *et al*., 2017) (Table S1). Around five seeds of each accession were directly sown on Jiffy pots (Jiffy Products International AS) and left for 48 hr at 4 °C for stratification. Afterwards, plants were grown under controlled conditions for 4 weeks (22 °C, 70% relative humidity [RH], 9 hr of light) before inoculation. The two homozygous *sds‐2* and *sds‐3* mutants (SAIL and FAG105 T‐DNA insertion mutants in Columbia‐0 [Col‐0] and Wassilewskija [Ws‐4] genetic backgrounds, respectively) were kindly provided by Raphaël Mercier (INRA, Versailles, France) (De Muyt *et al*., 2009). An altered expression of *SDS* in these two mutants was confirmed in De Muyt *et al*. (2009). The two null mutants were grown as described above.

### 4.2 Plant inoculation and phenotyping

Four‐week‐old plants were root‐inoculated with the *R. solanacearum* GMI1000. The *R. solanacearum* GMI1000 reference strain was grown in complete BG agar, supplemented with 6 ml of glucose (20%) and 1 ml of triphenyltetrazolium chloride (1%), and incubated at 28 °C for 48 hr then left at room temperature for 24 hr. One day before inoculation, one colony was grown in liquid BG medium and grown overnight at 28 °C under shaking. Plants were inoculated with a bacterial suspension at OD_600 nm_ between 0.8 and 1. Before inoculation, roots were cut with scissors 1 cm from the bottom of the Jiffy pot (Deslandes *et al*., 1998). This method gives the bacteria direct access to the xylem vessels. During inoculation, plants were soaked in a bacterial suspension at 10^7^ bacteria/ml for 15 min. Inoculated plants were incubated in growth chambers at 27 or 30 °C (75% RH, 12 hr light, 100 μmol⋅m^−2^⋅s^−1^). Disease symptoms were scored daily from 3 to 9 dai using a disease index scale from 0 to 4 as previously described (Deslandes *et al*., 1998) with the scores from 0 to 4 corresponding to healthy and fully wilted plants, respectively.

### 4.3 Natural variation of QDR in the TOU‐A population

#### 4.3.1 Experimental design

For each temperature treatment, 624 plants were used and arranged by following a randomized complete block design (RCBD) of three temporal experimental blocks. Each block was represented by two trays of 104 positions, corresponding to one replicate per accession (*n* = 192 accessions) and the susceptible Col‐0 accession was placed in the same three positions within each tray (*n* = 6). In each block, the remaining 10 positions in the trays were kept empty. Note that plants of the third block were not scored at 9 dai.

#### 4.3.2 Statistical analysis

For each temperature treatment, a mixed model (MIXED procedure in SAS v. 9.4; SAS Institute Inc.) was used to explore the natural genetic variation of the disease index at each time point of phenotyping, as follows:

(1) $${\text{disease index}}_{\mathrm{ijc}}=\;\mu \;+{\;\text{block}}_{i}+{\;\text{accession}}_{j}+{\;\text{covCol}}_{c}+\;\varepsilon_{\mathrm{ijc}}$$

where *µ* is the overall mean of the phenotypic data, “block” accounts for differences in microenvironmental conditions between the three experimental blocks, “accession” corresponds to the genetic differences among the TOU‐A natural accessions, covCol is a covariate accounting for tray effects within blocks (phenotypic mean of the three Col‐0 replicates per tray was used as a covariate), and “ε” is the residual term. The factor “block” was considered as a fixed factor and the factor “accession” as a random factor. The significance of the random effect was determined by likelihood ratio tests of model with and without this effect. Residuals were normally distributed so no transformation was applied on raw phenotypic data. For GWA mapping analyses, we used best linear unbiased predictors (BLUPs) obtained for each natural accession. Because *A. thaliana* is a highly selfing species (Platt *et al*., 2010), BLUPs correspond to genotypic values. Using a formula adapted from Gallais (1990), broad‐sense heritabilities (*H* ^2^) at each time point of phenotyping were estimated from the variance component estimates of the “block” and “accession” terms obtained with the VARCOMP procedure in SAS v. 9.4 (SAS Institute Inc.).

#### 4.3.3 GWA mapping with local score analysis

To fine map the genomic regions with additive effects associated with natural disease index variation at each time of phenotyping for each temperature treatment, a mixed model implemented in the software EMMAX was adopted (Efficient Mixed‐Model Association eXpedited; Kang *et al*., 2010). To control for the effect of population structure, we included as a covariate an identity‐by‐state kinship matrix *K* based on the 1,902,592 SNPs identified in the TOU‐A population (Frachon *et al*., 2017). Because rare alleles increase the rate of false positives when included in mixed models, we considered a threshold of minor allele relative frequency (MARF) >7% and ended up with 981,617 SNPs (Brachi *et al*., 2010; Kang *et al*., 2010). As previously described in Frachon *et al*. (2017), a threshold of 7% corresponds to the MARF value above which the *p* value distribution obtained from the mixed model is not dependent on MARF values in the TOU‐A population.

Thereafter, we implemented a local score approach on the set of *p* values provided by EMMAX. The local score allows detection of significant genomic segments by accumulating the statistical signals from contiguous markers such as SNPs (Fariello *et al*., 2017). In a given QTL region, the association signal, through the *p* values, will cumulate locally due to LD between SNPs, which will then increase the local score (Bonhomme *et al*., 2019). Briefly, a sequence of scores is calculated along the chromosome as *X_i_* = −log_10_(*p_i_*) − *ξ*, where *p_i_* is the *p* value of marker *i* and ξ is a tuning parameter with an optimal value that can be fixed at 2 or 3 in a GWAS context (Bonhomme *et al*., 2019). Then, finding segments that accumulate strong signals is equivalent to finding peaks along a Lindley process defined as *h_i_* = max(0, *h_i_*_−1_ + *X_i_*) along the chromosome, with *h*_0_ = 0. Significant SNP–phenotype associations were identified by estimating a chromosome‐wide significance threshold for each chromosome (Bonhomme *et al*., 2019).

#### 4.3.4 Detecting QTL epistasis

In order to detect epistatic interactions among our set of 14 top QTLs identified with additive effects by combining a GWA mapping approach with a local score analysis, we followed the procedure adopted in Brachi *et al*. (2015). We first identified within each QTL region the SNP with the highest association score estimated by EMMAX, hereafter named bait top SNP. For each of the 14 QTLs, we then computed LD estimates between the bait top SNP and all the other SNPs in the TOU‐A population. LD between two biallelic (homozygous) SNPs was calculated using the absolute value of *r* statistic (correlation coefficient) between two SNP genotype vectors. For each QTL, we obtained a distribution of LD estimates between the bait top SNP and the other 981,616 SNPs of the population (MARF > 7%). In order to exclude strong LD values due to physical proximity, SNPs located in a 100 kb window surrounding a bait top SNP were not included in the calculation. To estimate whether the bait top SNP of a given QTL (i.e., focal bait top SNP) was significantly in LD with the bait top SNPs of the remaining 13 QTLs, we estimated in the LD distribution (conditional on each focal bait top SNP) the quantile *q* for each bait top SNP of the 13 QTLs. To be conservative, an LD estimate between a focal bait top SNP and another bait top SNP was declared significant if (1 − *q*) < .01.

#### 4.3.5 Estimates of allelic effect

To display the allelic effect of the bait SNPs after controlling for the effects of population structure, BLUPs estimated from model (1) were adjusted by fitting them with a kinship matrix. Kinship‐adjusted BLUPs were computed under the *R* environment 3.6.1 (R_Core_Team, 2019). In order to avoid pseudoreplication due to the presence of SNPs in stretches of LD, we first pruned the SNP data set with the snpgdspLD command using the following parameters: ld.threshold = 0.8, slide.max.bp = 500, maf = 0.07 (“gdsfmt” and “SNPRelate” packages), leaving 365,952 SNPs for the estimation of the kinship matrix. The kinship matrix was then estimated using the popkin function (allowing missing data in the SNP matrix) in the popkin package, with the subpopulation vector set to NULL. Because the resulting matrix was not positive semi‐definite, the function make.positive.definite() from the package lqmm was used. Finally, the kinship‐adjusted BLUPs were calculated with the function kin.blup from package rrBLUP. Keeping the notations from model (1), the parameters were: accession as geno, disease index as pheno, the above‐mentioned kinship matrix as K, GAUSS = F, indicating that the genotypes are not independent and follow G = K V_G_, block as fixed effect and covCol as covariate. The kinship‐adjusted BLUPs were then extracted using the command $pred.

### 4.4 Analysis of the *SDS* candidate gene

For each temperature treatment, an experiment of 288 plants was set up according to a RCBD of three temporal experimental blocks. Each block was represented by one tray of 96 positions, corresponding to 24 replicates of each genotype, that is, the *sds‐2* and *sds‐3* mutants with their corresponding wild‐type background Col‐0 and Ws‐4, respectively.

For each temperature treatment, we tested whether each null mutant differs from its corresponding wild‐type background along the infection stages by using the following model (MIXED procedure in SASv. 9.4; SAS Institute Inc.):

(2) $${\text{disease index}}_{\mathrm{ij}}=\;\mu \;+{\;\text{block}}_{i}+{\;\text{genotype}}_{j}+{\;\text{block}}_{i}\;\times {\;\text{genotype}}_{j}+\;\varepsilon_{\mathrm{ij}}$$

where *µ* is the overall mean of the phenotypic data, “block” accounts for differences in microenvironmental conditions between the three experimental blocks, “genotype” corresponds to the genetic differences between the T‐DNA mutant and its corresponding wild‐type background, “block × genotype” accounts for variation between genotype differences among blocks, and “ε” is the residual term. All factors were considered as fixed.

## AUTHOR CONTRIBUTIONS

R.B. and F.R. supervised the project. N.A., R.B., and F.R. designed the experiments. N.A. conducted the phenotyping experiments. N.A. and F.R. analysed the phenotypic traits. F.R. performed the GWA mapping. M.B. performed the genome‐wide local score analysis. L.B. performed the LD analyses. H.D. estimated the allelic effects of the QTLs. N.A., L.D., R.B., and F.R. wrote the manuscript. All authors contributed to the revisions.

## Data Availability

The data that support the findings of this study are available from the corresponding author upon reasonable request.
