# Supplementary material for: A complex network of additive and epistatic quantitative trait loci underlies natural variation of Arabidopsis thaliana quantitative disease resistance to Ralstonia solanacearum under heat stress
Source: Mol Plant Pathol. 2020 Sep 11;21(11):1405–20. doi: 10.1111/mpp.12964 (PMC7548995; doi:10.1111/mpp.12964)

**Figure S1. Box-plots illustrating the additive effects of each top SNP located in five QTLs not involved in epistatic interactions as well as the epistatic effects between QTLS 12A and 12B**. Blue, green, orange and red boxes indicate that the QTLs are located on chromosome I, II, IV and V, respectively. White boxes illustrate epistatic interactions among QTLs.


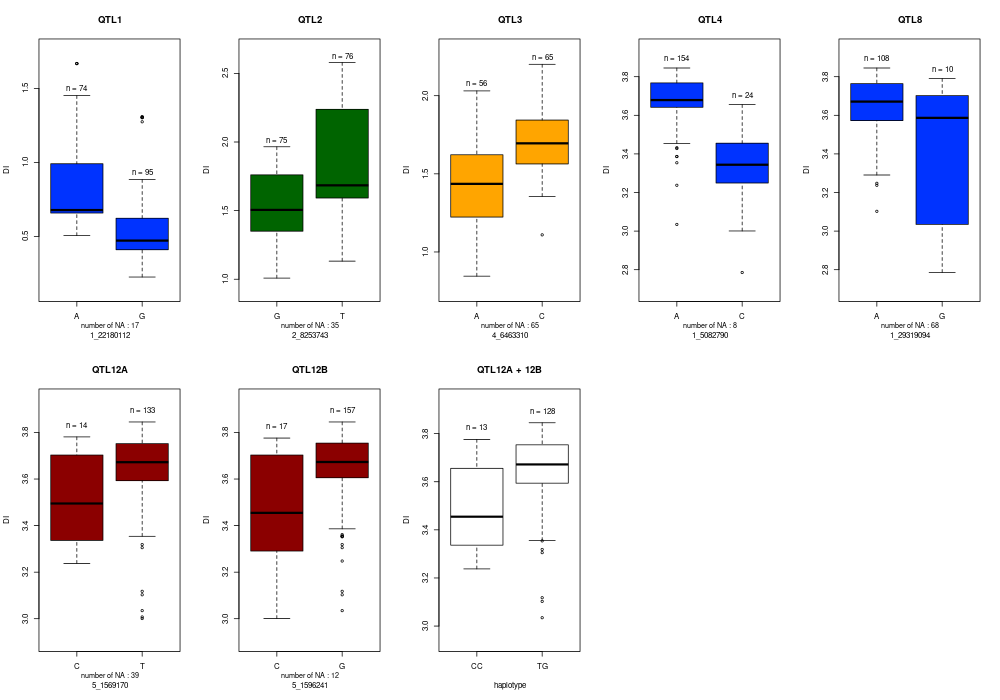

Supplement: Supplementary file 1 [file MPP-21-1405-s001.docx]
