# Supplementary material for: A complex network of additive and epistatic quantitative trait loci underlies natural variation of Arabidopsis thaliana quantitative disease resistance to Ralstonia solanacearum under heat stress
Source: Mol Plant Pathol. 2020 Sep 11;21(11):1405–20. doi: 10.1111/mpp.12964 (PMC7548995; doi:10.1111/mpp.12964)
Supplement: Supplementary file 2 [file MPP-21-1405-s002.docx]

**Figure S2. Detection of inter-QTLs epistasis for 11 out of the 14 QTLs with a Lindley process value above 10.** For each QTL, a genome-wide distribution (grey area) was established by calculating LD values between the bait top SNP and all the other SNPs across the genome (with the exception of the SNPs located in a 100kb window surrounding the bait top SNPs). Only SNPs with a MARF > 0.07 were considered. In addition, LD values (above 0.1) between the bait top SNP for the corresponding QTL and the bait top SNPs from the other QTLs are represented by arrows. The *x*-axis corresponds to the LD estimates expressed as the absolute value of the *r* correlation coefficient. The black line corresponds to the density curve.


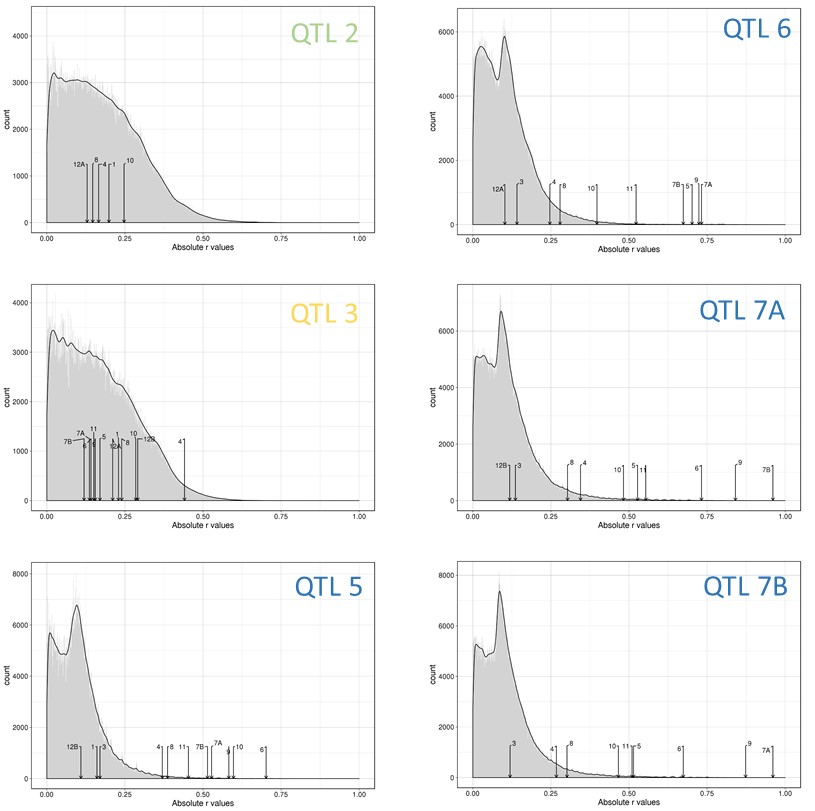


**Figure S2 (continued)**

**
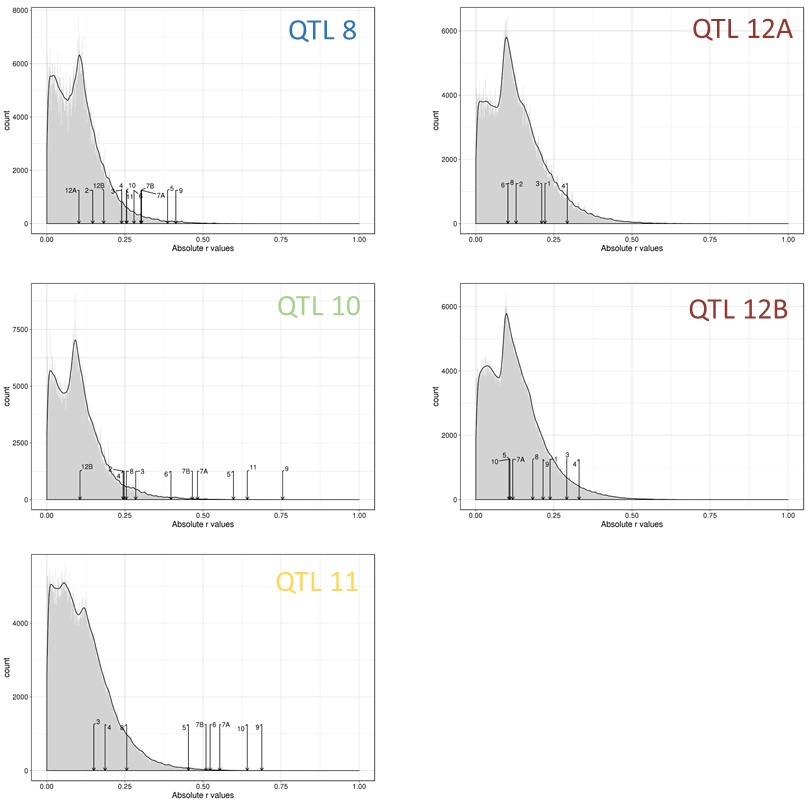
**
