# Supplementary material for: A complex network of additive and epistatic quantitative trait loci underlies natural variation of Arabidopsis thaliana quantitative disease resistance to Ralstonia solanacearum under heat stress
Source: Mol Plant Pathol. 2020 Sep 11;21(11):1405–20. doi: 10.1111/mpp.12964 (PMC7548995; doi:10.1111/mpp.12964)

**Figure S3. Growth dynamics and internal growth curve of GMI1000 reference strain at 27°C and 30°C. (a)** *in vitro* growth dynamics of GMI1000 reference strain. Bacterial cultures were grown at 27°C and 30°C in complete liquid medium starting from a single colony. The colored lines represent the mean of two independent biological repeats, each composed of ten technical repeats. Standard deviation at each time point is represented by a vertical bar. **(b)** Box-plot illustrating in planta bacterial growth at 27°C and 30°C using A. thaliana Col-0 susceptible accession plants. Each dot represents one of the 14 plants. *in planta* bacterial multiplication of the GMI1000 strain in Col-0 was significantly different between 27°C and 30°C (*F* = 5.69, *P* = 0.0250). * *P* < 0.05.


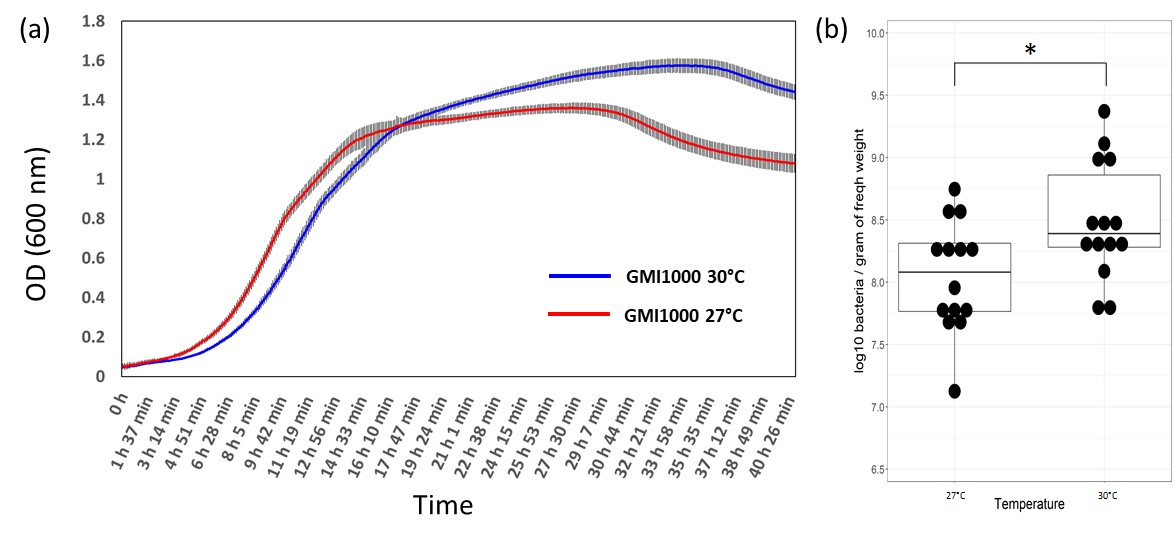

Supplement: Supplementary file 3 [file MPP-21-1405-s003.docx]
