# Supplementary material for: A complex network of additive and epistatic quantitative trait loci underlies natural variation of Arabidopsis thaliana quantitative disease resistance to Ralstonia solanacearum under heat stress
Source: Mol Plant Pathol. 2020 Sep 11;21(11):1405–20. doi: 10.1111/mpp.12964 (PMC7548995; doi:10.1111/mpp.12964)
Supplement: Supplementary file 4 [file MPP-21-1405-s004.docx]

**Table S1. List of the 192 natural accessions of the local TOU-A mapping population used in this study.** This list shows the ecotype ID and name of the accessions, as well as their germination status at 27°C and 30°C.

**Table S1 (continued)**
