# Supplementary material for: A complex network of additive and epistatic quantitative trait loci underlies natural variation of Arabidopsis thaliana quantitative disease resistance to Ralstonia solanacearum under heat stress
Source: Mol Plant Pathol. 2020 Sep 11;21(11):1405–20. doi: 10.1111/mpp.12964 (PMC7548995; doi:10.1111/mpp.12964)
Supplement: Supplementary file 5 [file MPP-21-1405-s005.docx]

**Table S2.** **Effects of knock-down of *SDS* expression at 3, 4, 5, 6, 7 and 10 dai against the *R. solanacearum* GMI1000 strain in two wild-type genetic backgrounds at 27°C and 30°C.**

*F*, *F* value resulting from the test of fixed effect. * *P* < 0.05; ***P* < 0.01; ****P* < 0.001, ne: not estimated due to the absence of phenotypic variation. Significant differences between each wild-type background and its corresponding mutant after a FDR correction are indicated in bold.
