# Supplementary material for: A complex network of additive and epistatic quantitative trait loci underlies natural variation of Arabidopsis thaliana quantitative disease resistance to Ralstonia solanacearum under heat stress
Source: Mol Plant Pathol. 2020 Sep 11;21(11):1405–20. doi: 10.1111/mpp.12964 (PMC7548995; doi:10.1111/mpp.12964)
Supplement: Supplementary file 6 [file MPP-21-1405-s006.docx]

**Supplementary method**

The GMI1000 *Ralstonia solanacearum* reference strain was grown in complete BG medium at 28°C (Plener *et al*., 2010). For agar plates, the complete medium was supplemented with d‐glucose (5 g.L^−1^) and triphenyl tetrazolium chloride (0.05 g.L^−1^). Bacteria was plated on solid complete medium and grown 48h at 28°C in order to have isolated colonies. Single colonies were grown overnight in complete liquid medium. Two ml of independent culture were centrifuged at 6000 rpm during 10 min and the pellet were resuspended in 2 mL of fresh complete BG liquid medium. An aliquot of 100 μl was used to determine the initial bacteria concentration, and bacteria were diluted to 5 × 10^7^ CFU.mL^−1^. 200 μl was used as inoculum. Bacterial growth was monitored using a microplate spectrophotometer (FLUOstar Omega, BMG Labtech, Germany) during 40 h at 27°C or 30°C under shaking at 700 rpm using a linear shaking mode.

For Arabidopsis plant assay, two biological replicates of 30 four-week old plants from the susceptible Col-0 accession were inoculated as described in Deslandes *et al.* (2003), at either 27°C or 30°C using a 10^8^ bacteria.mL^-1^ solution, without cutting the roots. Following inoculation, plants were immediately transferred in a growth chamber with the following conditions: 12h of light per day, RH 75% , 27°C day /26°C night , 30°C day/ 29°C night day regime. Disease development was monitored every day following the inoculation of *R. solanacearum* GMI1000 strain until 13 days. Internal Growth Curve was determined as described in Deslandes *et al.* (1998) by harvesting at each temperature plants with a mean disease index of 3 (i.e. plants with 75% of wilted leaves). We harvested 14 plants from the two biological replicates at both temperatures. Rosette were sterilized in 70% ethanol for 3 min and then rinsed three times in sterile water. Rosette were then dried and weighted, grinded and then resuspended in 2 mL sterile water. Bacterial concentration was determine by plating serial dilution of *R. solanaceraum* on SMSA medium (Elphinstone *et al.*, 1996).

**Plener L, Manfredi P, Valls M, Genin S. 2010.** PrhG, atranscriptional regulator responding to growth conditions, is involved in the control of the type III secretion system regulon in *Ralstonia solanacearum*. *Journal of Bacteriology* **192:** 1011–1019.

**Deslandes L, Olivier J, Peeters N, Feng DX, Khounlotham M, Boucher C, Somssich I, Genin S, Marco Y. 2003.** Physical interaction between RRS1-R, a protein conferring resistance to bacterial wilt, and PopP2, a type III effector targeted to the plant nucleus. . *Proceedings of the National Academy of Sciences of the United States of America* **100:** 8024–8029

**Deslandes L, Pileur F, Liaubet L, Camut S, Can C, Williams K, et al. 1998.** Genetic characterization of RRS1, a recessive locus in *Arabidopsis thaliana* that confers resistance to the bacterial soil borne pathogen *Ralstonia solanacearum*. *Molecular Plant Microbe Interactions* **11:** 659–667.

**Elphinstone JG, Hennessy J, Wilson JK, Stead DE. 1996.** Sensitivity of different methods for the detection of Ralstonia solanacearum in potato tuber extracts. EPPO Bulletin **26:** 663-678.
